# ChatGPT in Drug Discovery Process

**DOI:** 10.34172/apb.2024.007

**Published:** 2023-08-16

**Authors:** Ammu V.V.V. Ravi Kiran, Garikapati Kusuma Kumari, Praveen T. Krishnamurthy

**Affiliations:** Department of Pharmacology, JSS College of Pharmacy (JSS Academy of Higher Education & Research), Ooty, The Nilgiris, Tamil Nadu 643 001, India.

## Dear Editor,

 Over the decades, advancements in technological fields have greatly reshaped the drug development process in pharmaceutical companies. This drive in drug development process is due to an increase in computed generated prediction models and pre-screening of new chemical entities.^1^ In recent years, artificial intelligence such as ChatGPT has made its new entry, attracting many biopharmaceutical scientists, globally. ChatGPT is a large language model developed by OpenAI that is trained on a vast corpus of text data. It has been used in a variety of applications, including natural language processing, language translation, and text generation. One of the most promising applications of ChatGPT in the field of drug discovery is its ability to generate new chemical compounds. This is done by training the model on a dataset of known compounds and their properties.^2^ Once trained, the model can generate new chemical compounds that are similar to the compounds in the dataset. This can be useful in the drug discovery process as it can help to identify new compounds that have the potential to be developed into drugs. Another application of ChatGPT in drug discovery is its ability to predict the properties of new compounds. By training the model on a dataset of known compounds and their properties, it can predict the properties of new compounds that it has never seen before.^2^ This can be useful in the drug discovery process as it can help to identify compounds that have the potential to be developed into drugs.

 Additionally, ChatGPT can be used to assist in the process of literature mining, which is the process of extracting information from the scientific literature. This can be useful in the drug discovery process as it can help to identify new compounds and their properties, as well as help to identify potential drug targets.^2,3^ However, it should be noted that ChatGPT is a model trained on a large corpus of text data and its predictions should be considered in light of the current knowledge of the field and should be validated by experimental testing.^4^

 Furthermore, the drug discovery process is a complex and multi-disciplinary field, involving various steps such as target identification, lead generation, lead optimization, preclinical development, clinical development, and regulatory approval.^4,5^ Therefore, ChatGPT should be used as a complementary tool rather than a stand-alone solution.

 In conclusion, ChatGPT is a powerful tool that can assist in the drug discovery process by generating new chemical compounds, predicting the properties of new compounds, and assisting in literature mining. However, more research is needed to fully understand its capabilities and limitations, and it should be used in conjunction with other tools and techniques in the drug discovery process.

## Competing Interests

 None.

## Ethical Approval

 Not applicable.
